# Sensible

**Published:** 1883-04-20

**Authors:** 


### *SENSIBLE.*

There are some American physicians, says the Medical and Surgical Reporter, who write too much ; but the great majority do not write enough.

Medical journals are the vehicles of interchange of thought and experience between the members of the profession; and a physician should make it a rule always to report every case that may occur in his practice in which any phenomena may present themselves that he does not find recorded in his text-books.

But he should be short and explicit. Many an otherwise good communication has been rendered worthless by being too *long-winded;* the valuable points have been smothered in a lot of trash.

When a man feels sure that he has something *new* to report, he should reflect how he can say what he has to say in as few words as possible. He will thus prepare an article that will be readable as well as valuable, and will not encroach unnecessarily on the too limited space of a good journal.

Physicians should likewise cultivate the habit of independent thought and reasoning in their practice; and should not, as so many do, practice strictly according to the rules laid down in the books.

Short, practical communications are always of value, and the profession of our country should make it a rule to prepare them frequently.

